# Exploiting sorghum genetic diversity for enhanced aluminum tolerance: Allele mining based on the *Alt*_*SB*_ locus

**DOI:** 10.1038/s41598-018-27817-z

**Published:** 2018-07-04

**Authors:** Barbara Hufnagel, Claudia T. Guimaraes, Eric J. Craft, Jon E. Shaff, Robert E. Schaffert, Leon V. Kochian, Jurandir V. Magalhaes

**Affiliations:** 1 Embrapa Maize and Sorghum, Sete Lagoas, MG Brazil; 2 Departamento de Biologia Geral, Universidade Federal de Minas Gerais, Belo Horizonte, MG 31270-901 Brazil; 3 Robert W. Holley Center of Agriculture and health, USDA-ARS, Cornell University, Ithaca, New York, USA; 4 Global Institute for Food Security, University of Saskatchewan, Saskatoon, SK S7N 4J8 Canada; 5 Present Address: Centre National de la Recherche Scientifique, Biochimie et Physiologie Moléculaire des Plantes, Montpellier SupAgro, 2 Place Pierre Viala, 34060 Montpellier, France

## Abstract

Root damage due to aluminum (Al) toxicity restricts crop production on acidic soils, which are extensive in the tropics. The sorghum root Al-activated citrate transporter, SbMATE, underlies the Al tolerance locus, *Alt*_*SB*_, and increases grain yield under Al toxicity. Here, *Alt*_*SB*_ loci associated with Al tolerance were converted into *Amplification Refractory Mutation System* (ARMS) markers, which are cost effective and easy to use. A DNA pooling strategy allowed us to identify accessions harboring rare favorable *Alt*_*SB*_ alleles in three germplasm sets while greatly reducing genotyping needs. Population structure analysis revealed that favorable *Alt*_*SB*_ alleles are predominantly found in subpopulations enriched with guinea sorghums, supporting a possible Western African origin of *Alt*_*SB*_. The efficiency of allele mining in recovering Al tolerance accessions was the highest in the largest and highly diverse germplasm set, with a 10-fold reduction in the number of accessions that would need to be phenotyped in the absence of marker information. Finally, Al tolerant accessions were found to rely on *SbMATE* to exclude Al^3+^ from sensitive sites in the root apex. This study emphasizes gene-specific markers as important tools for efficiently mining useful rare alleles in diverse germplasm, bridging genetic resource conservation efforts and pre-breeding for Al tolerance.

## Introduction

Crop aluminum (Al) toxicity on acidic soils (soil pH ≤ 5), which comprise approximately 50% of the world’s arable lands^1^, severely reduces crop yields on a global scale. Because acid soils are prevalent in tropical and sub-tropical regions such as in Sub-Saharan Africa^2^, where sorghum is an important staple food crop for millions of poor rural people, Al tolerance is critical for food security. Aluminum ions are solubilized from clay minerals at acidic soil pH values as the rhizotoxic ionic form, Al^3+^, which damages root systems, thereby reducing root growth. As a consequence, water and nutrient uptake are constrained, leading to severe yield losses^3–6^.

Plant species exhibit extensive genetic variation for Al tolerance that is associated both with internal and external mechanisms of tolerance^5,7^. Some plant species can detoxify Al internally, via formation of non-toxic Al complexes with organic acids or other chelators, with subsequent sequestration of these complexes into the vacuoles^8,9^. The most widespread and studied Al tolerance mechanism takes place in the root tip apoplast and rhizosphere, where low molecular weight organic acids such as citrate and malate are released from the root apices via plasma membrane transporters. These organic acids chelate Al^3+^, forming non-toxic, Al-organic acid complexes, thereby protecting the root apex, which is particularly sensitive to Al^3+ ^^7,10^. Al-activated citrate and malate transporters belonging to the Aluminum-Activated Malate Transporter (ALMT) and the Multidrug and Toxic Compound Extrusion (MATE) families have been shown to mediate organic acid-based Al exclusion from the root apices, thus conferring Al tolerance in plant species. The first major Al tolerance genes belonging to these gene families were cloned in wheat (*TaALMT1*^11^), sorghum (*SbMATE*^12^) and barley (*HvAACT1*^13^). A number of homologs of these genes have been subsequently identified in other cultivated plant species including maize^14^, soybean^15^, rice^16^, rice bean^17^, wheat^18^, barley^19^, rye^20^, rapeseed^21^ and also in Arabidopsis^22,23^.

In sorghum, the *Alt*_*SB*_ locus on chromosome 3 controls a large fraction of the phenotypic variation for Al tolerance^24^. Positional cloning showed that *SbMATE* (GenBank accession EF611342), an Al-activated citrate transporter that is more highly expressed in root apices of Al tolerant accessions, underlies the *Alt*_*SB*_ locus. *SbMATE* is transcriptionally induced by Al^3+^ and mediates citrate release from root apices, thereby conferring sorghum Al tolerance^12^. Introgression of a single tolerant *SbMATE* allele has been recently shown to increase sorghum grain yield by ~0.6 ton ha^−1^ on an acid, Al toxic soil, both in inbred lines and in hybrids^25^. Based on the Carvalho *et al*. study^25^, in homozygosity, *SbMATE* increased grain yield by a striking 50% over the population mean with no yield penalty in the absence of Al toxicity. This makes *SbMATE* a major asset for sorghum production on acid soils.

Highly Al tolerant accessions occur at a low frequency in sorghum (0.05) and are not randomly distributed across the sorghum diversity *continuum*, being prevalent primarily in guinea and to a lesser extent in caudatum sorghums^26^. Therefore, provided the target Al tolerant alleles are present in a given sorghum germplasm, typically extensive Al tolerance phenotyping is necessary for identifying Al tolerance sources. Association mapping applied to the *Alt*_*SB*_ locus was used to identify SNP and indel loci either within or in close vicinity to *SbMATE* that were associated with Al tolerance^27^. In a sorghum association panel, 79% of the accessions harboring the A *SbMATE* allele at the intronic SNP locus, 6083, were either highly or intermediately Al tolerant. This contrasts with the phenotypic proportions for Al tolerance and sensitivity, where 79% of the accessions in the association panel were Al sensitive. These results indicate that allele mining strategies, which seek to identify novel, superior and beneficial alleles in potentially large germplasm or natural populations^28,29^, may be used to facilitate the identification of Al tolerance donors in sorghum.

Here we describe the development of an easy-to-use marker system based on *Amplification Refractory Mutation System* (ARMS) markers^30^ to tag *SbMATE* SNP loci previously shown to be associated with Al tolerance in a sorghum association panel. We validated those markers by allele mining in three different sorghum germplasm sets, including a panel comprising sorghum accessions adapted to West Africa. Finally, we characterized the physiological and molecular nature of the superior *Alt*_*SB*_ alleles that were identified, which are now available for breeders throughout the world to increase sorghum yields on acidic, Al toxic soils.

## Results

### *Alt*_*SB*_ marker development

**Figure 1:**
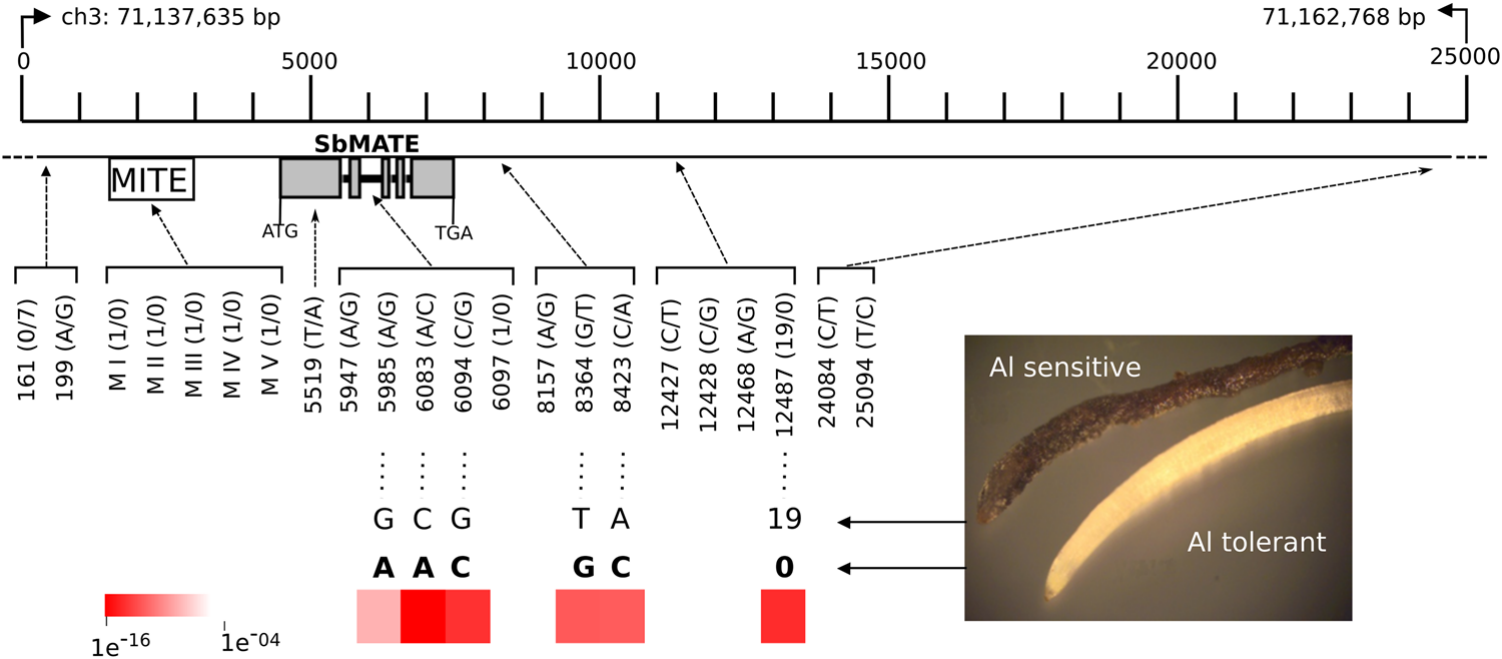
Position and association signals with Al tolerance of the loci used for marker development. The top bar depicts base pair positions in the context of the *Alt*_*SB*_ locus. The MITE transposon inserted in the promoter region of *SbMATE* and the *SbMATE* gene model are depicted (gray boxes indicate exons). Root apices of Al tolerant and Al sensitive lines, with the Al sensitive lines exhibiting typical high Al accumulation as revealed by hematoxylin staining^24^, are shown along with the respective *Alt*_*SB*_ alleles (alleles linked in coupling with Al tolerance are in bold type). Association analysis with Al tolerance was reported by Caniato *et al*.^27^ with a population structure (Q) + kinship (K) model and a heat map depicting the resulting association probabilities is shown for the selected loci (modified from Fig. 3 in Caniato *et al*.^27^).

We selected five SNPs and one indel loci that we have shown to have strong association with Al tolerance^27^ to use for marker development. Those are the SNP loci, 5985, 6083 and 6094, which are located within the second intron of *SbMATE*, and SNPs 8364 and 8423, which are found approximately 1 kb downstream of the *SbMATE* stop codon (Fig. 1). The indel locus, 12487, is located approximately 4 Kb downstream of 8364/8423.

**Figure 2:**
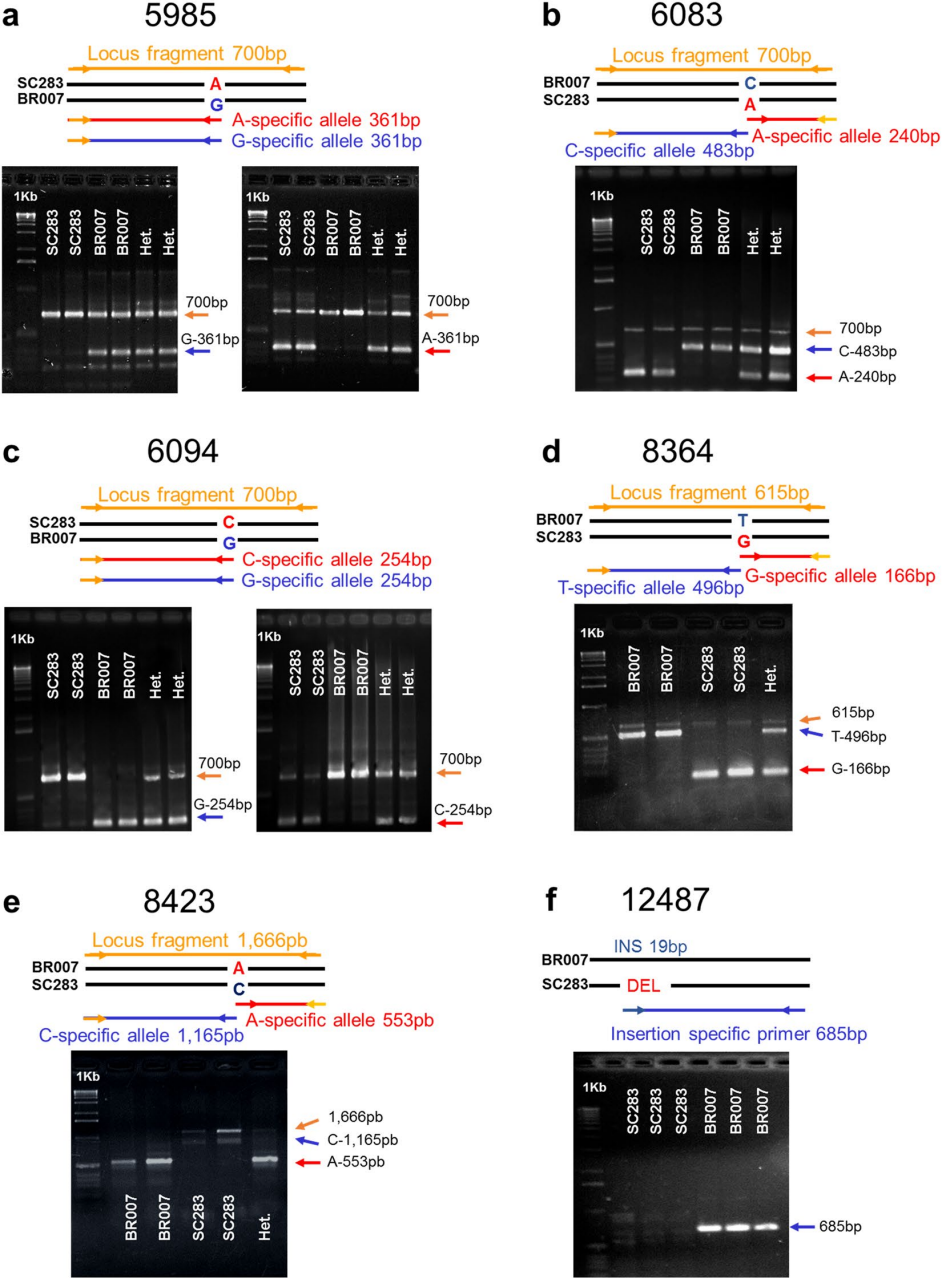
ARMS-PCR and Indel markers for *Alt*_*SB*_. Amplifications profiles are shown for two sorghum lines with different *Alt*_*SB*_ haplotypes, SC283 (Al tolerant) and BR007 (Al sensitive) as well as for a heterozygous individual. A 1 Kb molecular-weight size marker was loaded in the first well. The PCR products were resolved in 1.5% agarose gel. The amplification profiles of dominant (**a**,**c**), co-dominant (**b**,**d**,**e**) and the dominant indel (**f**) marker systems are shown. We are showing cropped gel images, which are presented either individually for each co-dominant marker (**b**,**d**,**e**) and for the indel marker (**f**) or separated by blank spaces in the case of each of the two alleles revealed by each dominant marker system (**a**,**c**).

Dominant (based on three primers) and four-primer, codominant ARMS-PCR^30^ marker systems were developed (Supplementary Table S1) for the SNP loci 5985 and 6094 (Fig. 2a,c) and for SNPs 6083, 8364, 8423 (Fig. 2b,d,e), respectively. For 6083 and 8423, confirmatory genotyping with the three-primer system was occasionally necessary. Amplification with all ARMS primers except for 8423 yielded a monomorphic band, which can thus be used as a positive PCR control as reported previously^30^. Insertion-specific primers were developed for the indel locus 12487 (Fig. 2f). The amplification profile for a dominant marker system developed for locus 5519 is shown in Supplementary Fig. S1. This marker tags a rare allele (A) specifically present in the highly Al tolerant line, SC566^31^, which was not used for allele mining in the present study due to its low frequency but can be used for a marker assisted backcross approach using SC566 as the Al tolerance donor.

### Pooling strategy

DNA pooling is an efficient approach for identifying low-frequency alleles, such as *Alt*_*SB*_ alleles that confer Al tolerance^27^, which minimizes the number of PCR reactions needed when large germplasm collections are to be screened. For efficient pooling, favorable alleles need to be detected even when they are present in much lower proportions compared to the alternative alleles. Optimum pool sizes were calculated based on the method described by Amos *et al*.^32^ and revised by Gastwirth^33^, using the allele frequencies reported in Caniato *et al*.^27^. Simulations based on population sizes ranging from 100 to 400 individuals indicated the optimum pool size to be four individuals per pool (Supplementary Table S2). Using this pool size for genotyping with marker 6083, pooling efficiency varied from 43.2% to 85.8% for a population size of 100 and 400 individuals, respectively (Supplementary Table S2).

**Figure 3:**
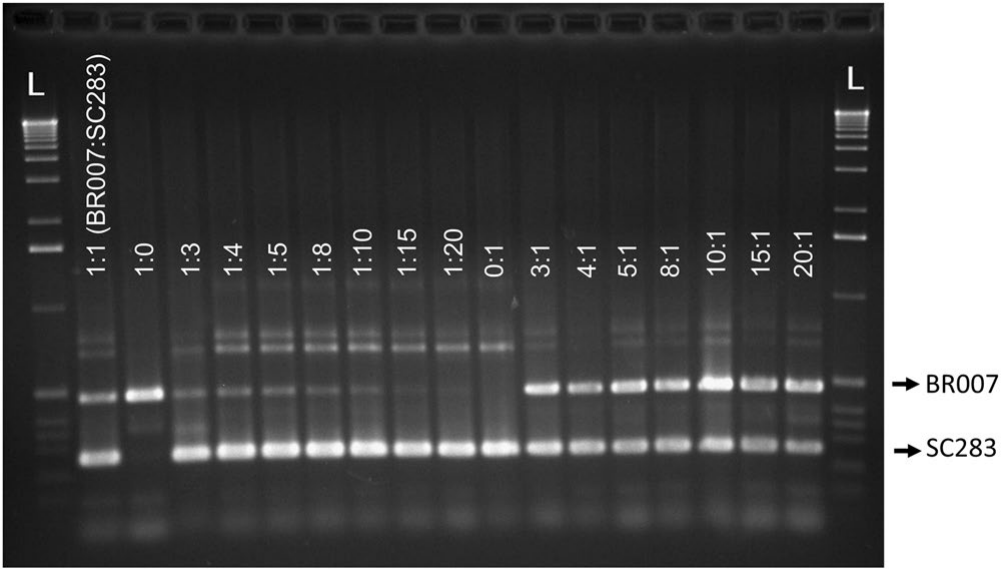
Detection sensitivity upon DNA pooling for the four-primer 6083 ARMS. DNA from the sorghum lines, BR007 (Al sensitive) and SC283 (Al tolerant) were mixed in equal (1:1) proportions (BR007:SC283) or so that the BR007 (1:3 to 1:20) or the SC283 (3:1 to 20:1) alleles were under-represented in the DNA pool at different proportions. Unmixed DNA from BR007 (1:0) and SC283 (0:1), which are homozygous for the unfavorable and favorable alleles, respectively, were included as controls. L = 1Kb ladder.

Next, a sensitivity test was performed for all marker loci by preparing mixtures with different proportions of each marker allele. The objective of this test was to confirm if the favorable allele of each marker would be detectable even under strong under-representation within a pool. Figure 3 shows a representative amplification profile for marker 6083. This marker was tested in samples obtained by pooling different proportions of genomic DNA of BR007 (Al sensitive) and SC283 (Al tolerant) in ratios varying from 1(BR007):0(SC283) to 1:20. The favorable, SC283 allele at 6083 was clearly detected even when the alternate BR007 allele was present at a 20-fold excess (20:1, Fig. 3), which indicates that the optimal pool size of four individuals determined for this marker (Supplementary Table S2) is adequate for proper detection of the target allele even when a single accession harboring the SC283 is present in the DNA pool. In contrast, amplification of the Al sensitive allele was strongest when only this allele was present in the pool and showed reduced efficiency even at the lowest degree of under-representation (1:3), although it was clearly detectable even at the 1:10 proportion. Because it is the Al tolerant allele that is rare compared to the Al sensitive allele, higher amplification efficiency of the former with the ARMS system is advantageous for allele mining strategies based on the 6083 marker to identify Al tolerant accessions. Reduced detection sensitivity for the favorable allele was observed only for marker 8364, which was therefore not deemed appropriate for use in DNA pooling strategies.

### Allele mining for aluminum tolerance based on *Alt*_*SB*_

The *Alt*_*SB*_ markers were used to identify accessions harboring Al tolerant alleles of *Alt*_*SB*_ in three different sorghum panels, designated based on their origins as members of the SAP (Sorghum Association Panel, with 377 accessions^34^), INRAN (*Institut National de la Recherche Agronomique du Niger* panel, with 164 accessions) and ICRISAT (International Crops Research Institute for the Semi-Arid Tropics panel, with 187 accessions). Upon genotyping, accessions harboring at least one favorable allele at the marker loci were phenotyped for Al tolerance based on Al inhibition of root growth in nutrient solution.

**Figure 4:**
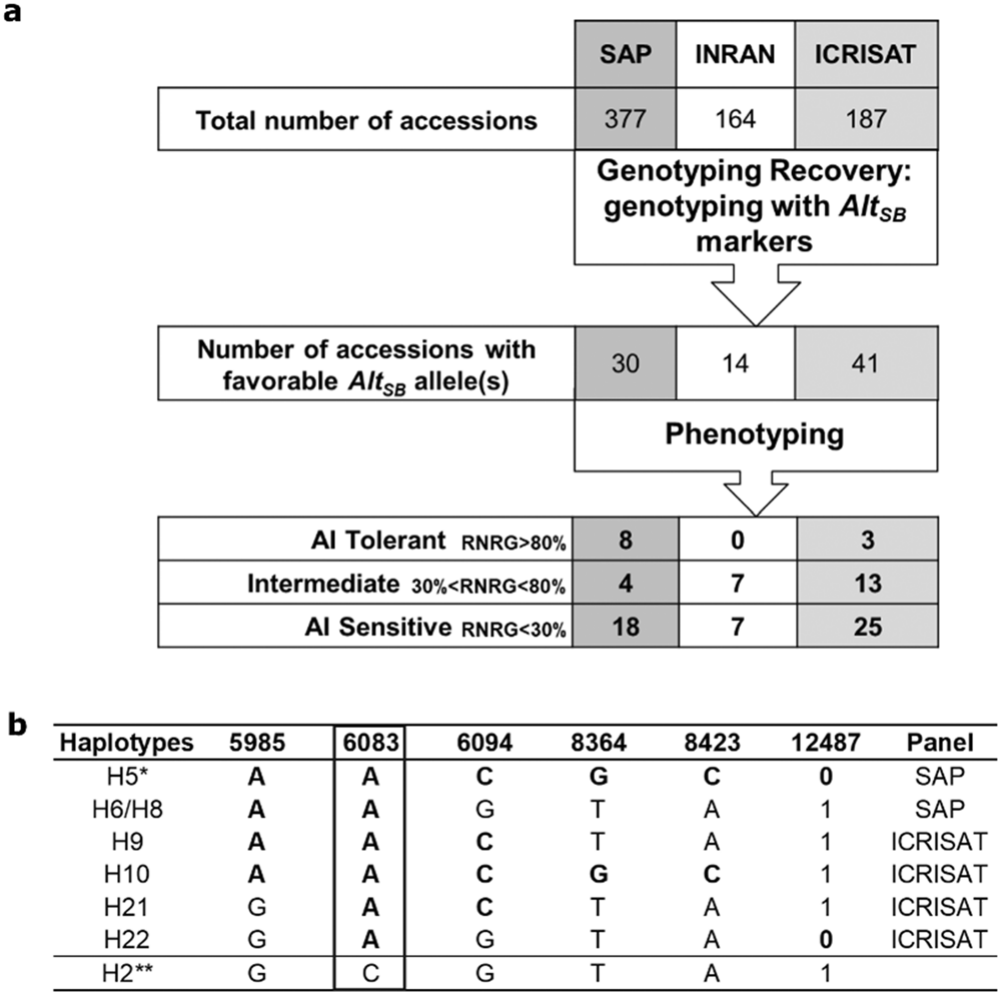
Allele mining in the SAP, INRAN and ICRISAT panels. (**a**) Diagram depicting the allele mining procedure in the SAP, INRAN and ICRISAT panels, which were used to identify accessions harboring at least one favorable *Alt*_*SB*_ allele at marker loci, 5985, 6083, 6094, 8364, 8423 and 12487. The distribution of those accessions in classes of Al tolerance is shown. (**b**) Haplotypes of Al tolerant accessions and the Al sensitive line, BR007 (H2), included as reference. Alleles in bold are linked in coupling with Al tolerance^27^. The reference Al tolerant and Al sensitive haplotypes from SC283 (H5) and BR007 (H2), which is possibly an ancestral haplotype^27^, are marked with single and double asterisks, respectively. Al tolerance was assessed via Al inhibition of root growth (relative net root growth, RNRG) in nutrient solution containing {27} µM Al^3+^ at pH 4.

Of the 377 accessions from the SAP panel, 30 possessed at least one favorable *Alt*_*SB*_ allele (Fig. 4a). Phenotypic assessment of Al tolerance showed that 60% (18) of those accessions were sensitive to Al (RNRG < 30%), while the remaining 40% were either intermediately tolerant to Al (30% < RNRG < 80%, 4 accessions) or Al tolerant (RNRG > 80%, 8 accessions). Fourteen INRAN accessions harbored favorable *Alt*_*SB*_ alleles (Fig. 4a) and those were equally split into intermediately tolerant and Al sensitive, with no Al tolerant accession present in this panel. The frequency of accessions with favorable *Alt*_*SB*_ alleles was similar in the SAP and INRAN panels (0.08) but much lower compared to the ICRISAT panel (0.22). Although 41 accessions with favorable alleles were identified in the ICRISAT panel, the majority of those were intermediately Al tolerant (13 accessions), whereas 3 highly Al tolerant accessions were found in that panel. These data indicate that the expression of the Al tolerance phenotype in accessions harboring favorable *Alt*_*SB*_ alleles is highly variable across panels in a manner that cannot be fully explained by differences in population size.

The only marker locus for which all highly Al tolerant accessions identified in the SAP and INRAN panels harbored only the favorable allele was the SNP locus, 6083 (Fig. 4b). Previously, 6083 was found to show the strongest statistical association with Al tolerance, increasing Al tolerance by a striking ~60% RNRG, and was the most effective for the identification of Al tolerant accessions in a 254-member sorghum association panel (Fig. 3 and Table 2 in^27^).

We compared the power of selection based solely on the favorable allele of the 6083 marker to selection based on the presence of at least one favorable *Alt*_*SB*_ allele considering all loci combined in identifying Al tolerant and intermediate accessions (Table 1). Selection with marker 6083 alone allowed us to recover thirty-four out of a total of thirty-five Al tolerant and intermediate accessions identified when the three panels were genotyped with all markers used for allele mining, at the same time reducing the number of Al sensitive accessions (Table 1). No other marker yielded the same efficiency in selecting Al tolerant and intermediate accessions compared to 6083 (Supplementary Table S3).

**Table 1:** Number of Al tolerant and intermediate (T + I), and Al sensitive (S) accessions with at least one favorable *Alt*_*SB*_ allele considering joint selection with all markers or harboring the favorable (A) allele at marker 6083.

Panel	Response to Al	Accessions with at least one favorable allele considering all markers	Marker 6083 (**A** allele)
SAP (377)	T + I	12	11
	S	18	16
INRAN (164)	T + I	7	7
	S	7	7
ICRISAT (187)	T + I	16	16
	S	25	25

The total number of accessions in each panel is shown after the panel name.

### Population structure and allele mining

**Figure 5:**
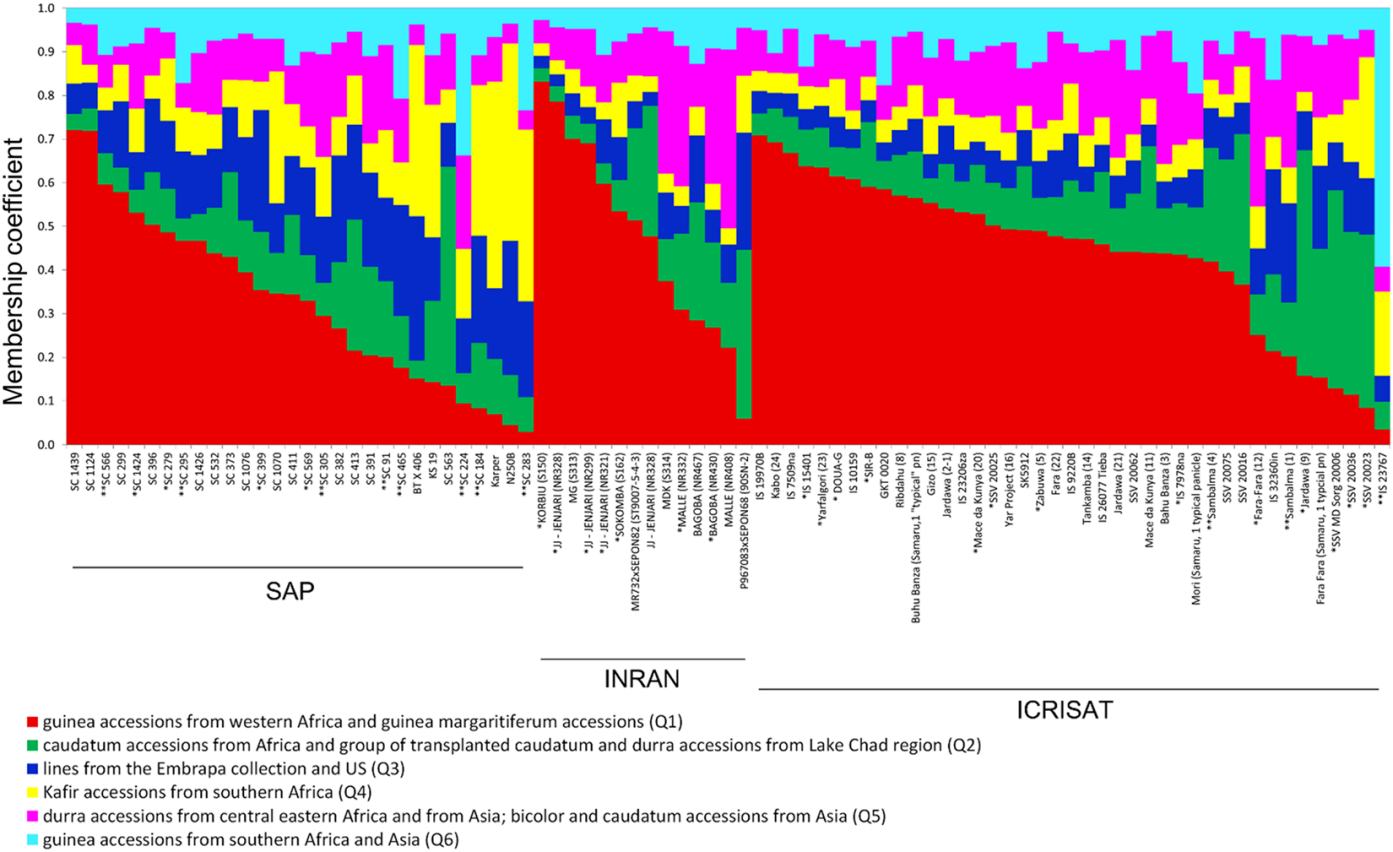
Subpopulation assignments for sorghum accessions with favorable *Alt*_*SB*_ alleles. The genotypic data for accessions with favorable *Alt*_*SB*_ alleles was integrated into the Caniato *et al*.^26^ dataset (Fig. S3 and Table S6 in^26^) by genotyping with the same set of SSR markers used in that study. The subpopulations are: Q1, guinea accessions from western Africa and guinea margaritiferum accessions; Q2, caudatum accessions from Africa and group of transplanted caudatum and durra accessions from Lake Chad region; Q3, lines from the Embrapa collection and US; Q4, kafir accessions from southern Africa; Q5, durra accessions from central eastern Africa and from Asia; bicolor and caudatum accessions from Asia; and Q6, guinea accessions from southern Africa and Asia. Double and single asterisks depict Al tolerant and intermediate individuals, respectively.

Previously, Caniato *et al*.^26^ identified six sorghum subpopulations featuring distinct racial and geographic origins using a 254-member sorghum association panel selected for maximum diversity. Therefore, we genotyped the 85 accessions harboring favorable *Alt*_*SB*_ alleles identified here by allele mining with the same markers used by Caniato *et al*.^26^ and assigned those accessions to the different subpopulations defined in that study based on their membership coefficients. Most of these accessions (76%) were assigned to subpopulations enriched for members in the guinea race (Fig. 5 and Supplementary Fig. S2). Within those, 62 accessions clustered with guinea margaritferum and guineas from Western Africa (subpopulation Q1) and 2 accessions were assigned to guineas from Southern Africa and Asia (Q6) (Fig. 5). Among those 64 accessions, six were Al tolerant (four in Q1 and two in Q6) whereas the other five Al tolerant accessions found across all panels (Supplementary Fig. S2) were assigned to subpopulation Q3 (one accession), which includes breeding lines used in Brazil and in the US, kafir accessions from southern Africa (Q4, 2 accessions), and durra, bicolor and caudatum accessions from eastern Africa and Asia (Q5, 2 accessions). The two Al tolerant accessions classified as kafir from southern Africa are the Al tolerance standard, SC283 and SC184. SC283, which is in fact a guinea type^24^, could not be clearly attributed to any single subpopulation, as it showed relationship (membership coefficients between 0.2 and 0.4) to Q4 (kafir accessions from southern Africa), Q3 (Brazil and US derivatives) and Q6 (guineas from southern Africa and Asia) as shown in Supplementary Table S4, suggesting that inter-racial hybridizations took place in the lineage that originated SC283. SC184, in turn, is classified in the Caffira subseries^35^, supporting its higher membership coefficient to subpopulation Q4. Except for that, considering all panels, the majority of highly Al tolerant accessions clustered with guinea types.

Among the intermediate accessions, there was again prevalence of membership to guineas from western Africa and guinea margaritiferum (87.5%, Q1, Supplementary Fig. S2). All four intermediate accessions from the SAP belong to Q1 as well as the majority of intermediate accessions in the INRAN and ICRISAT panels (5 and 8, respectively). The other intermediate accessions clustered in Q2, which includes caudatum types and durra, and in Q5, which, in addition to those races, also includes bicolor accessions. Eighty percent of the Al sensitive accessions harboring favorable *Alt*_*SB*_ alleles (40 accessions) belonged to subpopulation Q1. The subpopulation membership coefficients for the 85 accessions selected by allele mining are shown in Supplementary Table S4.

### Physiological and molecular characterization of the accessions identified by allele mining with *Alt*_*SB*_ markers

SNP and indel loci associated with Al tolerance were previously used to identify eight different *Alt*_*SB*_ haplotypes, H1–H8, in a sorghum association panel (Fig. 1 in^27^). Here we undertook a physiological and molecular characterization of Al tolerant sorghum accessions harboring different *Alt*_*SB*_ haplotypes. This analysis included haplotypes that were most frequently found in the Al tolerant accessions identified in the present study by allele mining in our largest germplasm set, the SAP (H5, H6/H8; Table S4).

**Figure 6:**
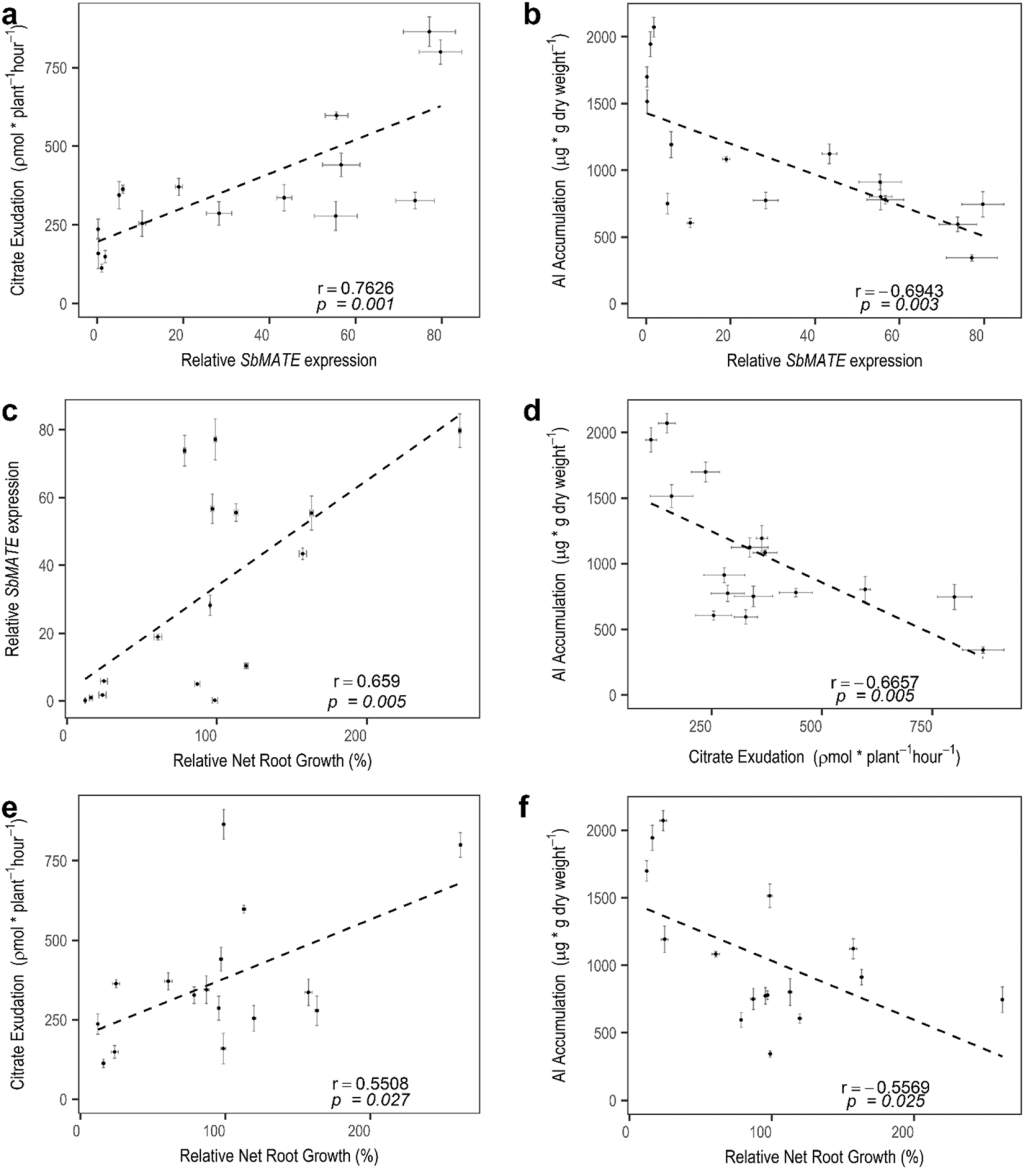
Correlation analysis between *SbMATE* expression and Al tolerance for sorghum accessions harboring different *Alt*_*SB*_ haplotypes. The Pearson correlation coefficient (r) and its associated *p*-value are shown. Standard error bars are shown. Correlation analysis was determined between the following physiological and molecular determinants of Al tolerance: *SbMATE* expression and citrate exudation (**a**), SbMATE expression and Al accumulation in root apices (**b**), *SbMATE* expression and Al tolerance measured by Relative Net Root Growth (RNRG) (**c**), citrate exudation and aluminum accumulation in root apices (**d**), Al tolerance and citrate exudation (**e**) and Al tolerance and Al accumulation in root apices (**f**). *SbMATE* expression was assessed^26^ by exposing sorghum accessions to {27} µM Al^3+^ for five days in nutrient solution at pH 4.0. For assessing citrate exudation, root exudates were collected for 6 hours in the presence of {27} µM Al^3+^. The first centimeter of the root was collected for assessing Al accumulation. Relative *SbMATE* expression was assessed by quantitative RT-PCR using the ΔΔCt method^50^.

A high and positive correlation coefficient was observed between *SbMATE* expression and citrate exudation (r = 0.76 p = 0.001, Fig. 6a), and *SbMATE* expression was also highly but negatively correlated with Al accumulation in root apices (r = −0.69 p = 0.003, Fig. 6b). These results, along with the positive correlation between *SbMATE* expression and Al tolerance (measured as relative net root growth, r = 0.66 p = 0.005, Fig. 6c) indicate that the accessions identified by allele mining effectively use *SbMATE*-dependent Al-activated citrate release to exclude Al from sensitive sites in the root apex as their main Al tolerance mechanism. Citrate release was highly and negatively correlated with Al accumulation (r = −0.66 p = 0.0005, Fig. 5d), indicating that *SbMATE* is indeed the major player in the Al exclusion mechanism taking place in those accessions. However, a slightly lower correlation coefficient of 0.55 between Al tolerance and citrate release/Al accumulation (Fig. 6e,f) suggest that some of these lines may also rely on auxiliary Al tolerance mechanism acting in concert with *SbMATE* to tolerate high levels of Al toxicity.

## Discussion

The prevalence of low-pH acidic soils in tropical and sub-tropical regions of the world^1^, coupled with the strong deleterious effect of Al toxicity on root development and crop production on these acid soils^36^, requires the efficient identification of Al tolerant genetic resources to ensure food security worldwide. The Al-activated root citrate transporter, SbMATE, which underlies the sorghum Al tolerance *Alt*_*SB*_ locus, has been shown to significantly increase grain yield under Al toxicity and, as such, is essential for sorghum production on acidic, Al toxic soils^25^. Efficient utilization of important genes for crop improvement while avoiding adoption of one or a few donors, which leads to an undesirable narrowing of the genetic basis, is a significant challenge in modern agriculture^37^. Hence, the need for developing a high throughput molecular breeding approach that could be flexibly adopted by plant breeders for sorghum production on acid soils motivated us to develop and validate *Alt*_*SB*_-specific markers. The resulting easy-to-use, low cost gene-specific markers can be used both for germplasm characterization via allele mining and for marker assisted introgression to improve sorghum Al tolerance.

The SNP and indel loci that were used to develop the *Alt*_*SB*_ markers were shown to be highly associated with Al tolerance in a sorghum association panel and some of them are possibly causative^12,27^. Marker loci based on functional polymorphisms or in tight linkage disequilibrium with causative variants, as is the case for the *Alt*_*SB*_ markers, offer greater flexibility for germplasm characterization in comparison to the traditional flanking marker approach. The latter requires the artificial creation of a high linkage disequilibrium context for detecting marker-trait associations, which is impractical when the final goal is to characterize large germplasm collections. If superior *Alt*_*SB*_ alleles can be identified directly in locally adapted accessions, the need for marker-assisted introgression of superior alleles from exotic donors is precluded, avoiding the time and cost associated with backcross breeding to recover adaptive traits.

Al tolerance has been shown to be a rare trait in sorghum, where only about 5% and 15% of the accessions show high and intermediate Al tolerance, respectively^26^. In addition, a population structure analysis showed that highly Al tolerant accessions are mostly found in subpopulations enriched for guinea and to a lesser extent caudatum sorghums. An analysis with *Alt*_*SB*_ haplotypes reconstructed from loci associated with Al tolerance showed that haplotypes with only Al sensitive alleles of *Alt*_*SB*_ are found at a frequency exceeding 0.8^27^. Conversely, the H5 haplotype, which harbors only alleles linked in coupling with Al tolerance, is likely a derived haplotype stemming from its lower frequency of 0.05, being prevalent in subpopulations enriched mostly in guinea types, which overlaps with the occurrence of the Al tolerance trait.

Our physiological and molecular analysis of different *Alt*_*SB*_ haplotypes was undertaken with sixteen accessions within which eleven were highly Al tolerant, one was intermediately tolerant, and four accessions were Al sensitive (based on relative net root growth at 5 days as shown in Supplementary Table S4 in^27^). The majority of the Al tolerant lines harbor haplotypes H5, H6 and H8^27^, which are the haplotypes found in all eight highly Al tolerant accessions identified by allele mining in the SAP panel (Supplementary Table S4). This analysis confirmed that high Al tolerance in these haplotypes results from allelic variation ultimately affecting *SbMATE* expression, with high *SbMATE* expression leading to higher organic acid release that acts to exclude Al^3+^ from sensitive sites in the root apex. The other three highly Al tolerant lines found in the ICRISAT panel possess the novel haplotypes, H9, H10 and H21, all of which, nonetheless, harbor the favorable, A allele, at the 6083 marker locus. We observed both here and previously in another germplasm set^27^ that maximum recovery of Al tolerant accessions can be obtained by selecting for the favorable allele of 6083 alone. Thus, it is likely that the allelic differences between H5, which harbor only Al tolerant alleles, and the H9, H10 and H21 haplotypes does not significantly change *SbMATE* expression and its function in providing Al tolerance via Al-activated citrate release into the rhizosphere.

Rare variants controlling important traits such as β-carotene content in maize grains have been reported and, as is the case for sorghum Al tolerance, are not randomly distributed in the species germplasm^38^. This indicates the need for designing specific and integrative strategies exploring complementarities between low and high LD contexts to maximize the chances of detecting rare variants, which is particularly challenging when based solely on genome wide scans with typically sub-optimal population sizes.

The majority (~73%) of the more Al tolerant accessions recovered in the present study by allele mining were clustered mostly in subpopulation Q1, which contains guinea types from Western Africa, supporting a Western-African origin of *Alt*_*SB*_ as previously hypothesized^26,27^. Within eighty-five accessions showing favorable *Alt*_*SB*_ alleles, we have been able to identify eight and three highly Al tolerant accessions in the SAP and ICRISAT panels, respectively. In the largest germplasm set, the SAP, among 30 accessions showing favorable *Alt*_*SB*_ alleles, eight (~27%) were highly tolerant to Al toxicity. Therefore, assuming the phenotypic frequency of high Al tolerance of 0.05 as previously reported^26,39^, *Alt*_*SB*_-based selection led to a 5.3-fold enrichment of Al tolerance in the SAP.

The usefulness of allele mining using *Alt*_*SB*_ markers compared to phenotypic selection alone to identify highly Al tolerance donors can be clearly perceived using our largest germplasm set, the SAP. In order to assess that we asked how many accessions of the 377-member SAP one would need to phenotype without marker information to rescue at least the same number of Al tolerant accessions, eight, that were identified within the thirty accessions selected by allele mining. This outcome can be modeled with a Poisson distribution with $$P=1-[(P(X=0)+$$$$P(X=1)+\ldots +P(X=m-1))|\lambda ]$$, where $$P$$ is the probability of success in rescuing at least a given number $$(m)$$ of Al tolerant accessions ($$X)$$, found at a frequency of 0.05. Based on sampling with $$\lambda =15\,(300\,\times \,0.05),\,$$in the absence of marker-based selection, 300 accessions, almost the entire panel, would need to be phenotyped to be 98% sure of identifying at least eight highly Al tolerant accessions. This contrasts with marker-based selection, whereby the same number of highly Al tolerant accessions were identified by screening only 30 accessions, which translates into a 10-fold reduction in the number of accessions needed to be phenotyped. Additionally, the DNA pooling strategy we employed significantly minimized the number of PCR reactions necessary for the detection of the rare mutations underlying the *Alt*_*SB*_ locus, which also improved the genotyping efficiency. This is demonstrated when we carried out allele mining in the SAP using the 6083 *Alt*_*SB*_ marker, where DNA pooling resulted in a 55% reduction in the number of samples to be genotyped. Because eight highly Al tolerant accessions were in fact identified in the SAP, allele mining with 6083 led to the efficient identification of Al tolerant donors with a great reduction in cost and time, which makes in-house genotyping with *Alt*_*SB*_ markers highly amenable to high throughput genotyping in large germplasm collections or breeding germplasm, with minimal infrastructure required.

Selection of favorable Al tolerant alleles using *Alt*_*SB*_ markers was not always associated with high Al tolerance, which we also observed previously^27^. In Melo *et al*.^31^ we had previously reported on an incomplete transfer of Al tolerance from parents to near isogenic lines, which was paralleled by a reduction in *SbMATE* expression^31,40^. Incomplete transfer of Al tolerance has been previously observed also in wheat^41^ indicating the importance of genetic background effects in the expression of the Al tolerance phenotype (reviewed by^42^). Therefore, the identification of Al sensitive lines showing Al tolerant haplotypes is likely due to the action of auxiliary loci influencing *SbMATE* expression in *trans*, the impact of which on Al tolerance is variable, depending on the magnitude of the *cis* effects controlled by elements located within the *Alt*_*SB*_ locus^31^.

The largest germplasm set used for allele mining, the SAP, is a diverse panel assembled to maximize geographic and racial diversity, including many guinea and caudatum types^43^. Thirty-four percent of the accessions in the SAP were assigned to guinea and/or caudatum subpopulations, 22% were assigned to subpopulations including guineas, caudatums and other races, and 44% clustered in subpopulations including other morphological races such as kafir and durra (Supplementary Table 1 in^43^). Therefore, it is not surprising that the most effective recovery of Al tolerant accessions was obtained using the SAP, since it includes the sorghum gene pool where Al tolerance is most prevalent. More than half of the accessions in the ICRISAT panel are also guinea types (Supplementary Table S4 and^44^), which are commonly grown in West Africa. Comparing the ICRISAT and the SAP panel, we were successful in identifying Al tolerant accessions in the ICRISAT panel but at a lower efficiency compared to the SAP (8 and 3 Al tolerant accessions were identified in the SAP and ICRISAT panels, respectively). However, the number of usable, Al intermediate donors was even higher in the ICRISAT panel. It is interesting that despite the smaller size of the ICRISAT panel (about half the number of accessions in the SAP), more accessions with favorable *Alt*_*SB*_ alleles were identified in the ICRISAT panel (41 *vs*. 30, Fig. 4), which possibly is due to a higher prevalence of guinea types in the ICRISAT panel compared to the SAP. It is thus possible that the highest population size and broader overall diversity of the SAP acted to minimize, at least to some extent, genetic background effects that appear to have a more prominent role in reducing Al tolerance in the ICRISAT panel.

We have only identified accessions with intermediate Al tolerance in the INRAN panel, which contained accessions collected only in Niger, encompassing different morphological races. Only 14 accessions with favorable *Alt*_*SB*_ alleles were found in the INRAN panel. With such a small space for Al tolerance to express itself over a context where genetic background effects are important, high Al tolerance in this germplasm or others where non-guinea/caudatum sorghums prevail would need to be introgressed from exotic sources.

To face the challenge of feeding the estimated world population of seven billion people by the year 2050, it is absolutely essential to sustainably improve crop yields on more marginal, stress-prone lands, such as in Sub-Saharan Africa. This study addresses this topic via exploiting and linking the cloning of *SbMATE*, which underlies the *Alt*_*SB*_ locus and has a strong beneficial impact on grain yield on acidic, Al toxic soils, with efficient germplasm characterization and marker-assisted selection strategies. Allele mining and marker assisted selection with gene-specific *Alt*_*SB*_ markers has great potential for improving food security on acid soils that comprise up to 40% of the world’s potentially arable lands^1^, while at the same time broadening the genetic basis of sorghum breeding programs targeting acid soil regions throughout the world.

## Materials and Methods

### Primer design and PCR analyses

Six SNP and one 19 bp indel loci located within the *Alt*_*SB*_ locus and previously shown to be associated with Al tolerance in a 254-member association panel^27^ were used for marker development for allele mining purposes. We designed a dominant marker system for the 19 bp indel polymorphism, where one of the primers anneals to the indel region so that the presence of a 685 bp PCR product tags the insertion allele. Marker conversion for the SNP loci was undertaken based on the ARMS-PCR strategy^30^. Briefly, this method employs primers flanking the target polymorphism either simultaneously, with two internal, allele-specific primers (codominant, four-primer system) or with each of the allele-specific primers being assayed in separate PCR reactions (dominant, three-primer ARMS). The last base in the 3′ end of the allele-specific primers is complementary to each SNP allele and, to increase specificity, a deliberate mismatch was introduced at position −2 or −3 from the 3′ terminus. Primers were designed using the primer design computer program, BatchPrimer3^45^, available at http://wheat.pw.usda.gov/demos/BatchPrimer3, by limiting the size ratio of the allelic bands to 1.1–1.5 fold, CG content between 20 to 80% and primer melting temperature (Tm) differing by at most 5 °C. Default settings were used for the other parameters.

The sorghum lines, BR007 (Al sensitive) and SC283 (Al tolerant), polymorphic for the selected SNP and indel loci (Table S4 in^27^), were used for marker validation. PCR reactions for the indel polymorphism were performed in a total volume of 20 µL with 2.0 pmol of each primer, 0.2 mM dNTP, 1 U of Taq polymerase, 20 mM Tris–HCl (pH 8.4), 50 mM KCl; 2 mM MgCl_2_ and 30 ng of genomic DNA. PCR reactions for the SNP markers were carried out in a 13 µL total volume with 2.0 pmol of each outer primer, 10 pmol of each inner (allele-specific) primer, 1X Go Taq® Green Master Mix (Promega, Foster City, CA) and 30 ng of genomic DNA. PCR reactions for SNP 8364 contained 1.2 pmol of the inner primers. Amplifications proceeded with an initial denaturation step of 94 °C for 4 min followed by 35 cycles at 94 °C for 1 min, 1 min of annealing at specific temperatures for each assay as detailed in Supplementary Table S1, 72 °C for 1 min followed by a final extension step at 72 °C for 7 min. The amplification products were separated by electrophoresis in a 1.5% agarose gel. Primer sequences are shown in Supplementary Table S1.

### Pooling strategy

We estimated the optimal pool size that would ensure the identification of low frequency Al tolerance alleles with minimum genotyping using the method described by Amos *et al*.^32^ and revised in^33^. First, we simulated population sizes ranging from 100 to 400 individuals and calculated the pool sizes and associated expected pooling efficiency (1 − F) as follows.

Taking the favorable allele frequency as π and *n* as the number of individuals, for *s* sized pools, the probability γ that at least one accession within a pool has the target allele is given by γ = 1 − (1 − π)^*s*^. The expected number of PCR reactions needed to identify all mutations in a given population is E(Y) = (*n*/*s*) + nγ. If the ratio E(Y):*n*, F, is smaller than 1, the pooling strategy leads to a reduction in the number of PCR reactions^33^. The pooling efficiency is given by 1 − F, where F is the ratio between γ and n.

To determine the sensitivity of the different marker assays to identify the desired allele with a given pool size, we mixed in different proportions genomic DNA of two contrasting lines for *Alt*_*SB*_, BR007 and SC283, and verified if the allele of interest could be visually detected.

### Allele mining based on *Alt*_*SB*_

Three sorghum panels were used for allele mining based on the *Alt*_*SB*_ locus: 1) The Sorghum Association Panel (SAP) panel is composed of 377 sorghum accessions including both tropical converted and breeding accessions^43^ and is available at the USDA-ARS – Germplasm Resources Information (ARS – GRIN, https://www.ars-grin.gov/); 2) the INRAN panel was provided by the *Institut National de la Recherche Agronomique du Niger* (INRAN, BP 429 Niamey, Niger) and has 164 accessions, of which 142 are landraces and 22 are breeding lines and 3) the ICRISAT panel was provided by the International Crops Research Institute for the Semi-Arid Tropics (ICRISAT, BP 320 Bamako, Mali) and has 187 accessions. Genomic DNA was isolated from 500 mg of leaf tissue from a total of 728 accessions using the protocol described by^46^ and PCR reactions for the *Alt*_*SB*_ markers were performed as described in the section primer design and PCR analyses. Within each panel, the total number of accessions was divided into pools, which contained DNA of four accessions per pool. Then, 100 ng of pooled genomic DNA was used for genotyping with the *Alt*_*SB*_ markers. Accessions within each “positive” pool, that is, pools containing favorable *Alt*_*SB*_ alleles, were then genotyped individually.

### Assessment of Al tolerance in nutrient solution

The sorghum accessions with favorable *Alt*_*SB*_ alleles were analyzed for Al tolerance in nutrient solutions containing either 0 or 148 µM Al^3+^, which correspond to free Al^3+^ activities of {0} and {27} µM (values inside brackets indicate Al^3+^ activity estimated with the speciation software, GEOCHEM-EZ^47^). The experiments consisted of a completely randomized design with two replications and seven plants per replication.

Hydroponic assessment of Al tolerance was undertaken as described in^40^. Briefly, seeds of each genotype were germinated for four days and seedlings were transferred to containers with nutrient solution lacking Al at pH 4.0, which were placed in a growth chamber with 27 °C day and 20 °C night temperatures, light intensity of 330 mmol photons m^−2^ s^−1^, under a 12 h photoperiod. After 24 h of acclimation, the *i*nitial *l*ength of each seedling’s root growing in *c*ontrol solution (*ilc*) was measured. The solution was then replaced by nutrient solution of identical composition but containing either no Al or {27} µM Al^3+^ supplied as AlK(SO_4_)_2_.12H_2_O. *F*inal root *l*engths under *Al* treatment (*flAl*) or *c*ontrol solution (*flc*) were obtained after five days of exposure to Al. For each line, mean values of relative net root growth (RNRG) were estimated by dividing net root growth under Al treatment (*flAl* - *ilc*) by net root growth without Al (*flc* - *ilc*) and expressed in percentage. We classified each sorghum accession for Al tolerance as described previously by Caniato *et al*.^26^: Al sensitive (RNRG < 30%), intermediately tolerant (30% < RNRG < 80%, designated intermediate) and Al tolerant (RNRG > 80%).

### Population structure of sorghum accessions carrying superior *Alt*_*SB*_ alleles

Accessions with favorable *Alt*_*SB*_ alleles were assessed for population structure. Caniato *et al*.^26^ previously assessed population structure of 254 sorghum accessions representative of the diversity present in cultivated sorghum. The same 38 simple sequence repeat (SSR) markers used in^26^ were used for genotyping the accessions identified in the present study by *Alt*_*SB*_-based allele mining. These SSR markers belong to an SSR kit (http://sorghum.cirad.fr/SSR_kit/) developed within the Generation Challenge Programme (GCP) and are evenly distributed across the sorghum genome. Fragment sizes were determined based on migration relative to an internal size standard using the GeneMapper 3.5 software. Allele sizes obtained for 10 control lines were compared to the expected allele sizes posted on http://sorghum.cirad.fr/SSR_kit/alleles.html and a correction factor for each marker was imposed to normalize allele sizes so that accessions containing favorable *Alt*_*SB*_ alleles identified in the present study could be integrated into the^26^ database. A Bayesian cluster analysis implemented in the software STRUCTURE^48^ was used to estimate population structure. This analysis was undertaken based on previous information on population structure in sorghum described in^26^, which revealed six subpopulations featuring distinct racial and geographic origins. The admixture model with correlated allele frequencies was adopted, with burn-in length 100,000 and 100,000 run length, for k equal 6.

### Physiological and Molecular Analyses

We selected 16 sorghum accessions representing different *Alt*_*SB*_ haplotypes for further physiological and molecular analyses related to *SbMATE*-mediated Al exclusion from root apices. The 16 sorghum accessions included the most Al tolerant accessions in^27^ and the Al sensitive lines, BR007 and BR012. The final set encompassed six of the eight *Alt*_*SB*_ haplotypes described in^27^ and included H5 and H6/H8, which are the haplotypes most frequently found in the Al tolerant accessions identified by allele mining in our largest germplasm set, the SAP (H5, H6/H8; Supplementary Table S4). Accession designations with the respective haplotypes (H) are: BR007 (H2), BR012 (H1), SC112 (H2), IS29691 (H2), IS21519 (H5), IS25077 (H4), IS10801 (H5), SC283 (H5), IS14351 (H5), IS23142 (H1), IS26457 (H6), IS26554 (H6), SC549 (H6), SC566 (H8), 9929030 (H8) and SC175 (H2), which were characterized for Al tolerance in nutrient solution as described previously.

### Root Organic Acid Exudation

Seeds were germinated as described above. Roots of seven uniform seedlings of each accession were inserted through holes (2 mm) drilled on the bottom of polyethylene cups, which were transferred to containers filled with 8 L of nutrient solution lacking Al (pH 4.0). These containers were then placed in a growth chamber with 27 °C day and 20 °C night temperatures, light intensity of 330 mmol photons m^−2^ s^−1^, and a 12 h photoperiod. After 24 h of acclimation, the solution was replaced by nutrient solution of identical composition but containing either no Al or {27} µM Al^3+^ supplied as AlK(SO_4_)_2_.12H_2_O. After five days of exposure to Al, the plastics cups were transferred to falcon tubes filled with 45 mL of a simple salt solution containing 4.3 mM CaCl_2_ pH 4.5 with or without {27} µM of Al^3+^. Exudate collection was allowed to proceed for 6 h for each experimental unit consisting of seven plants per accession, with three replications. After 6 hours, the exudate solutions were collected and passed through an OnGuard II Ag anionic silver chromatograph column (Dionex, http://www.dionex.com.br/) and then treated with a Dowex® 50WX8 cationic resin (Sigma Aldrich, http://www.dionex.com.br/). Subsequently, 1 mL sub-samples were lyophilized and resuspended in 0.1 mL ultrapure water. Organic acid analysis was performed using a capillary electrophoresis system as described in^49^. Root apices were collected for assessing root tip Al accumulation as described below.

### Al Accumulation in Root Apices

Seed germination and cultivation in hydroponics were as described above for quantitation of Al tolerance. For assessing root tip Al accumulation, each experimental unit consisted of seven plants per accession, with 3 replications. After five days of exposure to nutrient solution containing {27} µM Al^3^ at pH 4.0, seedling roots were washed with 8 L of ultra-pure water for 15 min, under aeration. A second washing period of 25 min was allowed to proceed with fresh ultra-pure water. The first centimeter of the primary roots was collected and oven-dried at 55 °C overnight. Dry weights were determined using a microgram balance (MT2; Mettler, Greifensee, Switzerland). Dry samples were digested with 100 µL of 70% (w/v) perchloric acid, resuspended in 2 mL of 0.5% (w/v) nitric acid and analyzed using an inductively coupled argon plasma emission spectrometer (Model 51000, Perkin-Elmer/Sciex, Norwalk, CT).

### *SbMATE* expression profile

Sorghum seedlings were grown following the same procedures used for assessing Al tolerance in nutrient solution containing {27} µM Al^3+^. Each experimental unit (accession) consisted of the first centimeter of root apices collected from 28 intact plants, five days after Al^3+^ exposure. These 28 plants per genotype were divided into four sets (seven plants per set) and each set was randomized inside a growth chamber. Total RNA was isolated from tissue samples using the RNeasy Plant Mini Kit (Qiagen, Valencia, CA) and 10 U of DNase I (RNase free) from the same manufacturer was added to each sample following incubation at room temperature for 15 min. First-strand cDNA was synthesized using 2 mg of total RNA with the High-Capacity cDNA Reverse Transcription Kit (Applied Biosystems, Foster City, CA). *SbMATE* transcripts were quantified with using the TaqMan Gene Expression kit on the ABI Prism 7500 Real Time PCR System (Applied Biosystems, Foster City, CA). A series of cDNA dilutions were used for making standard curves both for *SbMATE* transcripts and for 18S RNA which was used as the internal reference. Then, the selected dilution for specific cDNA samples (10 ng for SbMATE transcripts and 0.01 ng for 18S RNA) were used as real-time PCR templates to quantify relative transcript levels following the conditions recommended by the manufacturer. The forward (F) and reverse (R) primers, as well as the probe sequences are F: 59-CAG CCATTGCCCATGTTCTTT-39, R: 59-ACCAGCTTGCTCAGCATTATCA-39 and Probe: 6FAM- CCCAGTACCTGATAACGC-TAMRA. Levels of expression for endogenous 18S RNA were determined using TaqMan Ribosomal RNA Control Reagents (Applied Biosystems, Foster City, CA). Distilled water or products of room temperature reactions without reverse transcriptase were used as negative controls. *SbMATE* transcript levels were normalized to endogenous 18S RNA and *SbMATE* expression relative to that in the Al sensitive accession, BR012, was calculated. Three technical reps were used. The experiment was repeated twice with similar results.

### Data availability

The datasets generated during and/or analyzed during the current study are available from the corresponding author upon reasonable request.

## Electronic supplementary material

Supplementary information

Table S1

Table S4
