# Supplementary material for: Exploiting sorghum genetic diversity for enhanced aluminum tolerance: Allele mining based on the AltSB locus
Source: Sci Rep. 2018 Jul 4;8:10094. doi: 10.1038/s41598-018-27817-z (PMC6031643; doi:10.1038/s41598-018-27817-z)
Supplement: Supplementary file 1 — Supplementary information [file 41598_2018_27817_MOESM1_ESM.pdf]

Supporting Information

Exploiting sorghum genetic diversity for enhanced aluminum tolerance: Allele mining based on the Alt_{SB} locus

Barbara Hufnagel[1,2,3], Claudia T. Guimaraes[1,2], Eric J. Craft[4], Jon E. Shaff[4], Robert E. Schaffert[1], Leon V. Kochian[5], Jurandir V. Magalhaes[1,2,*]

[1] Embrapa Maize and Sorghum, Sete Lagoas, MG, Brazil

[2] Departamento de Biologia Geral, Universidade Federal de Minas Gerais, Belo Horizonte - MG, 31270-901

[3] Present address: Centre National de la Recherche Scientifique, Biochimie et Physiologie Moléculaire des Plantes, Montpellier SupAgro, 2 Place Pierre Viala, 34060 Montpellier, France

[4] Robert W. Holley Center of Agriculture and health, USDA-ARS, Cornell University, Ithaca, New York, U.S.

[5] Global Institute for Food Security, University of Saskatchewan, Saskatoon, SK S7N 4J8, Canada

*jurandir.magalhaes@embrapa.br

The following pages include:

Supplementary Figure S1

Supplementary Figure S2

Supplementary Table S2

Supplementary Table S3

Supplementary Figure S1

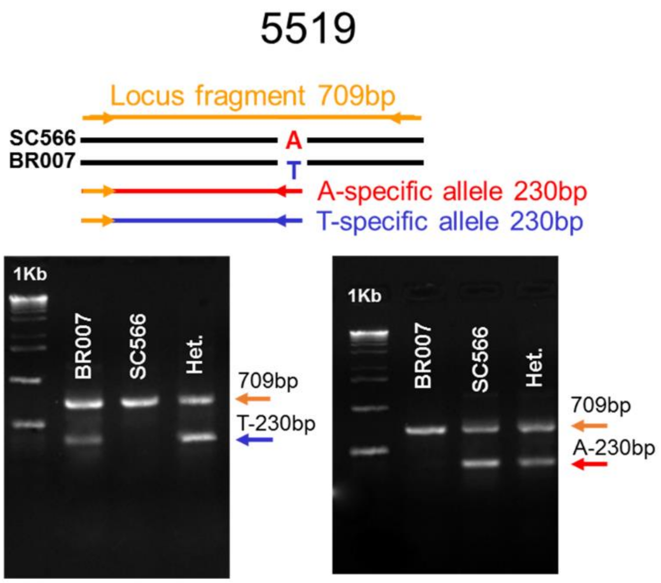

Supplementary Figure S1. ARMS-PCR marker for 5519 locus. The amplifications profile is shown for two sorghum lines with different *Alt*$_{SB}$ haplotypes, SC566 (Al tolerant) and BR007 (Al sensitive), and for a heterozygous individual. A 1 Kb molecular-weight size marker was loaded in the first well. The PCR products were resolved in 1.5% agarose gels. We are showing cropped images for the amplification profile of each allele.

Supplementary Figure S2

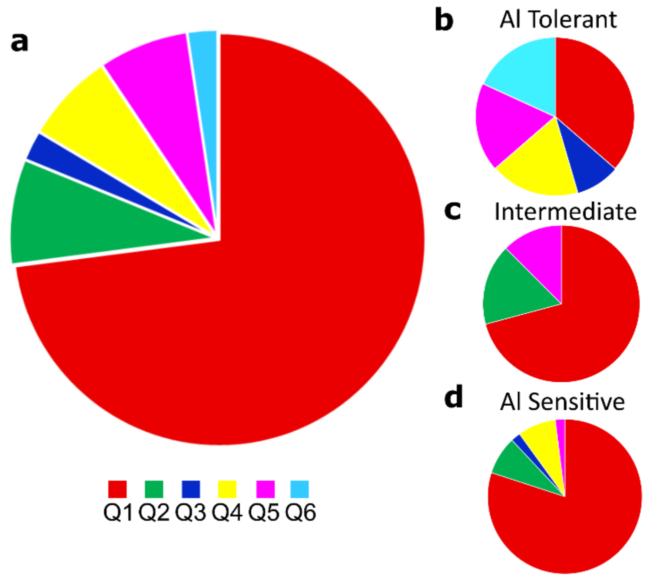

Supplementary Figure S2. Distribution of sorghum accessions with favorable *Alt$_{SB}$* alleles with respect to Al tolerance and subpopulation assignments. The subpopulations are: Q1, guinea accessions from western Africa and guinea margaritiferum accessions; Q2, caudatum accessions from Africa and the group of transplanted caudatum and durra accessions from Lake Chad region; Q3, lines from the Embrapa collection and US; Q4, kafir accessions from southern Africa; Q5, durra accessions from central eastern Africa and from Asia, bicolor and caudatum accessions from Asia; and Q6, guinea accessions from southern Africa and Asia. (a) All accessions with favorable *Alt$_{SB}$* alleles. (b) Al tolerant accessions (RNRG > 80%). (c) Accessions intermediately tolerant to Al (30% < RNRG < 80%). (d) Al sensitive accessions (RNRG < 30%).

Supplementary Table S2. DNA pooling efficiency. The values correspond to efficiency (1-F) with varying population sizes (n) and number of individuals per pool (S), simulated considering the frequency of the favorable allele. The maximum values for each condition are in bold.

Formulae

$\gamma = 1-(1-\pi)^s$	γ = probability that at least one pool member has the allele
$E(Y) = (n/S) + n\gamma$	S = pool size
$F = Y/n$	1-F = Pooling method efficiency

Polymorphism	Frequency (π)	S	Population Size (n)			
			100	150	200	400
5985	0.1345	3	**0.3150**	0.3150	0.3150	0.3150
		4	0.3111	**0.5408**	**0.6556**	**0.8278**
		5	0.2857	0.5238	0.6428	0.8214
		6	0.2537	0.5024	0.6268	0.8134
		7	0.2209	0.4806	0.6105	0.8052
		⋮				
		20	0.0056	0.3371	0.5028	0.7514
6083	0.0913		100	150	200	400
		3	0.4170	0.4170	0.4170	0.4170
		4	**0.4318**	**0.6212**	**0.7159**	**0.8580**
		5	0.4196	0.6131	0.3869	0.6131
		6	0.3964	0.5976	0.4024	0.5976
		7	0.3688	0.5792	0.4208	0.5792
		⋮				
		20	0.0974	0.3982	0.5487	0.7743
6094	0.0613		100	150	200	400
		3	0.4938	0.4938	0.4938	0.4938
		4	0.5264	0.6843	0.7632	0.8816
		5	**0.5288**	**0.6859**	**0.7644**	**0.8822**
		6	0.5175	0.6783	0.7587	0.8794
		7	0.4994	0.6662	0.7497	0.8748
		⋮				
		20	0.2322	0.4881	0.6161	0.8080

Supplementary Table S3: Phenotypic characterization for Al tolerance of lines harboring favorable alleles jointly at all marker loci or for each marker locus.

Panel		Number of accessions with at least one favorable allele considering all markers	Locus					
			5985	6083	6094	8364	8423	12487
	Alleles	-	**A**/G	**A**/C	**C**/G	**G**/T	**C**/A	**0**/19
IGD (377)	Tolerant + Intermediate	12	11	11	6	-	-	8
	Sensitive	18	9	17	11	-	-	6
INRAN (164)	Tolerant + Intermediate	7	7	7	7	5	7	2
	Sensitive	7	7	7	7	6	6	1
ICRISAT (187)	Tolerant + Intermediate	16	14	16	13	7	7	2
	Sensitive	25	20	25	23	10	11	11

In bold are the least frequent alleles, which are linked in coupling with Al tolerance. The total number of accessions in each panel are shown in parenthesis.
